# Enable a Facile Size Re-distribution of MBE-Grown Ga-Droplets via In Situ Pulsed Laser Shooting

**DOI:** 10.1186/s11671-021-03583-2

**Published:** 2021-08-04

**Authors:** Biao Geng, Zhenwu Shi, Chen Chen, Wei Zhang, Linyun Yang, Changwei Deng, Xinning Yang, Lili Miao, Changsi Peng

**Affiliations:** 1 School of Optoelectronic Science and Engineering and Collaborative Innovation Center of Suzhou Nano Science and Technology, Soochow University, Suzhou, 215006 China; 2 Key Lab of Advanced Optical Manufacturing Technologies of Jiangsu Province and Key Lab of Modern Optical Technologies of Education Ministry of China, Soochow University, Suzhou, 215006 China; 3 Institute of Research for Applicable Computing, University of Bedfordshire, Park Square, Luton, LU1 3JU UK; 4 AVIC Huadong Photo-electronics Co., Ltd, Wuhu, 241002 China

**Keywords:** Ga-droplet, In-situ pulsed laser, Thermal expansion, Thermal evaporation, Molecular beam epitaxy

## Abstract

A MBE-prepared Gallium (Ga)-droplet surface on GaAs (001) substrate is in situ irradiated by a single shot of UV pulsed laser. It demonstrates that laser shooting can facilely re-adjust the size of Ga-droplet and a special Ga-droplet of extremely broad size-distribution with width from 16 to 230 nm and height from 1 to 42 nm are successfully obtained. Due to the energetic inhomogeneity across the laser spot, the modification of droplet as a function of irradiation intensity (*IRIT*) can be straightly investigated on one sample and the correlated mechanisms are clarified. Systematically, the laser resizing can be perceived as: for low irradiation level, laser heating only expands droplets to make mergences among them, so in this stage, the droplet size distribution is solely shifted to the large side; for high irradiation level, laser irradiation not only causes thermal expansion but also thermal evaporation of Ga atom which makes the size-shift move to both sides. All of these size-shifts on Ga-droplets can be strongly controlled by applying different laser IRIT that enables a more designable droplet epitaxy in the future.

## Introduction

Currently with the increasing development of both fundamental physics and practical application, it is of great demands for people to achieve various devices. It has been widely demonstrated that various devices and structures can be constructed by applying of metallic nanoparticles [1–5]. As an important representative, droplet epitaxy which is based on metallic droplet (nanoparticles) has continuously attracted worldwide research interests and efforts since it is proposed by Koguchi et al*.* [6] in 1991 because it can nearly cover all kinds of low-dimensional nanostructures, including but not limited to quantum dots [7, 8], quantum rings [9–11] and quantum wires [12, 13]. Especially recently, some very peculiar structures of quantum-dot pairs [14, 15], quantum dot molecules [16, 17], double-rings [18] and multiple concentric rings [19, 20] are also successfully realized by droplet epitaxy. Generally, droplet epitaxy usually combines two steps, i.e., pre-formation of metallic droplet and subsequent crystallization [21, 22]. The size-control of droplets during the step of droplet formation is a key point for the whole droplet epitaxy since it not only determines the final size of quantum structure directly, but also defines what kind of nanostructure the droplets will target to become. For instance, a fast switch between quantum dots and quantum rings can be sensitively triggered by tuning the droplet size and the aforementioned multiple concentric rings is exclusively built on Ga-droplets of considerably huge size. As is well reported, temperature is the most essential factor for adjusting the size of droplets, in order to enlarge the droplet, the temperature must increase [23, 24]. Typically, Fuster et al*.* has increased the temperature to as high as 500 °C to successfully obtain a huge Ga-droplet with 45 nm in height and 240 nm in width [25]. However, increasing temperature will intensify the droplet etching into substrate drastically [26–29]. By this kind of nano-drill, the elements of droplets will be consumed before the subsequent crystallization and also a parasitic structure of nano-hole will develop beneath the droplet which may pollute the target quantum structure. Zh. M. Wang et al*.* has proven that the Ga-droplets could totally disappear and be replaced by volcano-like nano-holes only after annealing at 500 °C for 80 s without Arsenic (As) supplying [30]. Obviously, to raise temperature may destroy the droplets, but to push them growing bigger makes people has to do so, it`s an irreconcilable contradiction in the traditional droplet epitaxy. Hence, it makes great significances to find a technology, with temperature-independence, to modify the droplet size.

In this paper, Ga-droplets, with an original morphology of density: 4.1 × 10^10^/cm^2^, width: 37–65 nm and height: 4–9 nm, were produced on GaAs (001) substrate (Sub) through MBE and then immediately we used a UV pulsed laser to in situ shoot the as-prepared surface. Impressively, the laser shooting behaves a good modification of the size of droplets and the involved principle of the resizing from the *LIR* is systematically presented as well. After irradiation, the height and width of the droplets broaden to a range of 1–42 nm and 16–230 nm, respectively, i.e., we have successfully achieved extreme huge droplets with width as long as 230 nm and height as high as 42 nm directly at a very low temperature of 180 °C. So a technology for resizing the droplets with both safety and efficiency is reported here. This must contribute great freedom of size-control to the current droplet epitaxy and make it more feasible and flexible.

## Experimental Methods

**Fig. 1:**
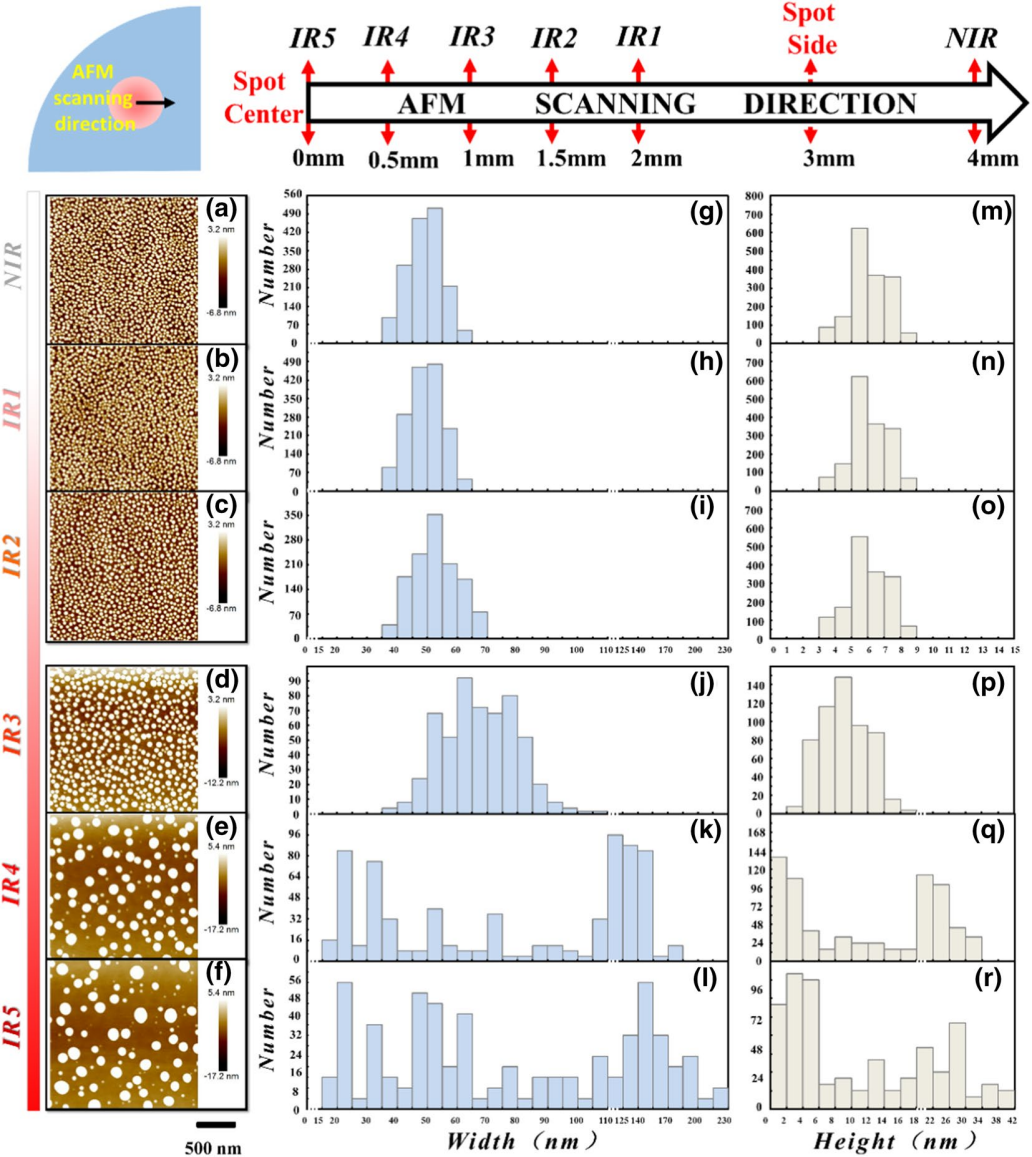
AFM morphology results of the droplets in **a** *NIR* and **b**–**f** *IR1-IR5*; the corresponding histograms of width and height distribution respectively in (g and m) *NIR* and (**h**−**l** and **n**−**r**) *IR1-IR5*; the top-draw shows the exact positions of *NIR* and *IR1-IR5* related to the laser spot

The experiments were performed on a special-designed MBE equipped with the laser viewport to in situ introduce pulsed laser beam into the chamber. At present, this prototype system only installs three source cells of Indium (In), Ga and As. The growth temperature is monitored by the pyrometer which is calibrated. For monitoring the growth, the reflection high energy electron diffraction is also comprised. First, a deoxidized GaAs (001) 2-inch quarter Sub was coated by a 300 nm GaAs buffer layer at 600 °C and the BEP of As_2_ is set as 7.6 × 10^−6^ Torr. Then, the As valve was full-closed and the Sub temperature was set down to 400 °C temporally to wait the excess As atoms being sufficiently captured by the liquid nitrogen cold trap and meanwhile to avoid the absorption of As on the surface. Until the As-ambient pressure was reduced to about 1.2 × 10^−9^ Torr that is nearly the same as the best pressure ((9.5 ~ 11) × 10^−10^ Torr) which can be obtained before growth to avoid the residual As_2_, the Sub temperature was furtherly decreased to 180 °C to form the droplets respectively with Ga growth rate of 0.168 ML/s and total deposition thickness of 4 MLs. As soon as the Ga-droplet growth was finished, the sample was in situ irradiated by only single shot of mono-beam of a frequency tripled neodymium yttrium aluminum garnet laser (wavelength:355 nm/pulse duration: 10 ns) with an energy of 35 mJ. After irradiation, the sample was immediately taken out to undergo the surface morphology test by AFM in tapping mode. Because the laser spot (6 mm/Diameter) is much smaller than the 1/4 2-inch Sub, both non-irradiated region (*NIR*) and irradiated region (*IR*) can be put together to compare. For the *IR*, due to the laser spot has a Gaussian-like profiled intensity distribution, the morphology evolution of the droplet as a function of *IRIT* can be all at once observed on this sample. So in the following discussion, five representative locations, defined as irradiation-1 (*IR1*) to irradiation-5 (*IR5*) in the *IRIT* order of *E*_*IR1*_ < *E*_*IR2*_ < *E*_*IR3*_ < *E*_*IR4*_ < *E*_*IR5*_, were selected from the *IR* for analysis and the exact positions of them related to the laser spot are marked in the top-draw of Fig. 1. As is shown, the position of *IR5* is corresponding to the center of the laser spot (marked as 0-position), then we linearly scanned rightward, after each movement of 0.5 mm, an AFM image was taken (corresponding to *IR4-IR1* in sequence). At last, we completely moved outside of the spot and took the AFM image defined as *NIR* (i.e., the original morphology of as-prepared Ga-droplets).

## Results and Discussion

Figure 1a–f present the AFM morphology results of the droplets in *NIR* and *IR1-IR5*, respectively. (g-l) and (m-r) are the corresponding histograms of width and height distribution. Since the droplets were fabricated at temperature as low as 180 °C, in *NIR* (Fig. 1a), the original density reaches to as high as 4.1 × 10^10^/cm^2^ and the width and height are both typically Gaussian distributed with dominant mode of 45–55 nm and 4–8 nm (respectively shown in Fig. 1g, m). The maximum and minimum sizes are corresponding ~ 65 nm wide/ ~ 9 nm high and ~ 37 nm wide/ ~ 4 nm high. The droplets in *IR1* (Fig. 1b) look very like with the *NIR*. There are no distinct changes can be distinguished in comparison of either Fig. 1h and (g) or (n) and (m). The droplets in *IR1* have the same maximum and minimum size with the *NIR*. While in *IR2* (Fig. 1c) and *IR3* (Fig. 1d), the droplet size begins to be modified by the laser shooting. Some enlarged droplets emerge on the surface with a reduction of the density. Especially for *IR3*, the droplets beyond the former maximum width (65 nm) have accounted to a proportion of 55% (Fig. 1j) and corresponding 37% for the proportion beyond the former maximum height (Fig. 1p). At the same time, the total density has reduced to only 1/3 of the original density. Overall, after the laser resizing, the size distribution of droplets in either of *IR2* and *IR3* is solely shifted to the large side, i.e., no droplets on the small side of the original distribution in *NIR* are observed. However, for the droplets in *IR4* and *IR5*, the distributions not only shift to the large side but also extend to the small side: Fig. 1e, f display the results of *IR4* and *IR5*, with continued reduction of the density, it is clear to see in Fig. 1k–l and q–r that the droplet size distributions are furtherly shifted to the large side. Particularly in *IR5*, the maximum droplet (width: 230 nm/height: 42 nm) is nearly four times larger than the maximum one (width: 65 nm/height: 9 nm) in *NIR* and such a large size is not reported elsewhere at so low a temperature. Besides, a few small droplets beneath the original minimum size are also generated and some of them even are ultra-mini only with width of 16 nm and height of 1 nm. So, the evolution of the laser modification of the Ga-droplets with *IRIT* is completely observed and it well demonstrates the laser shooting can facilely resize the Ga-droplets.

**Fig. 2:**
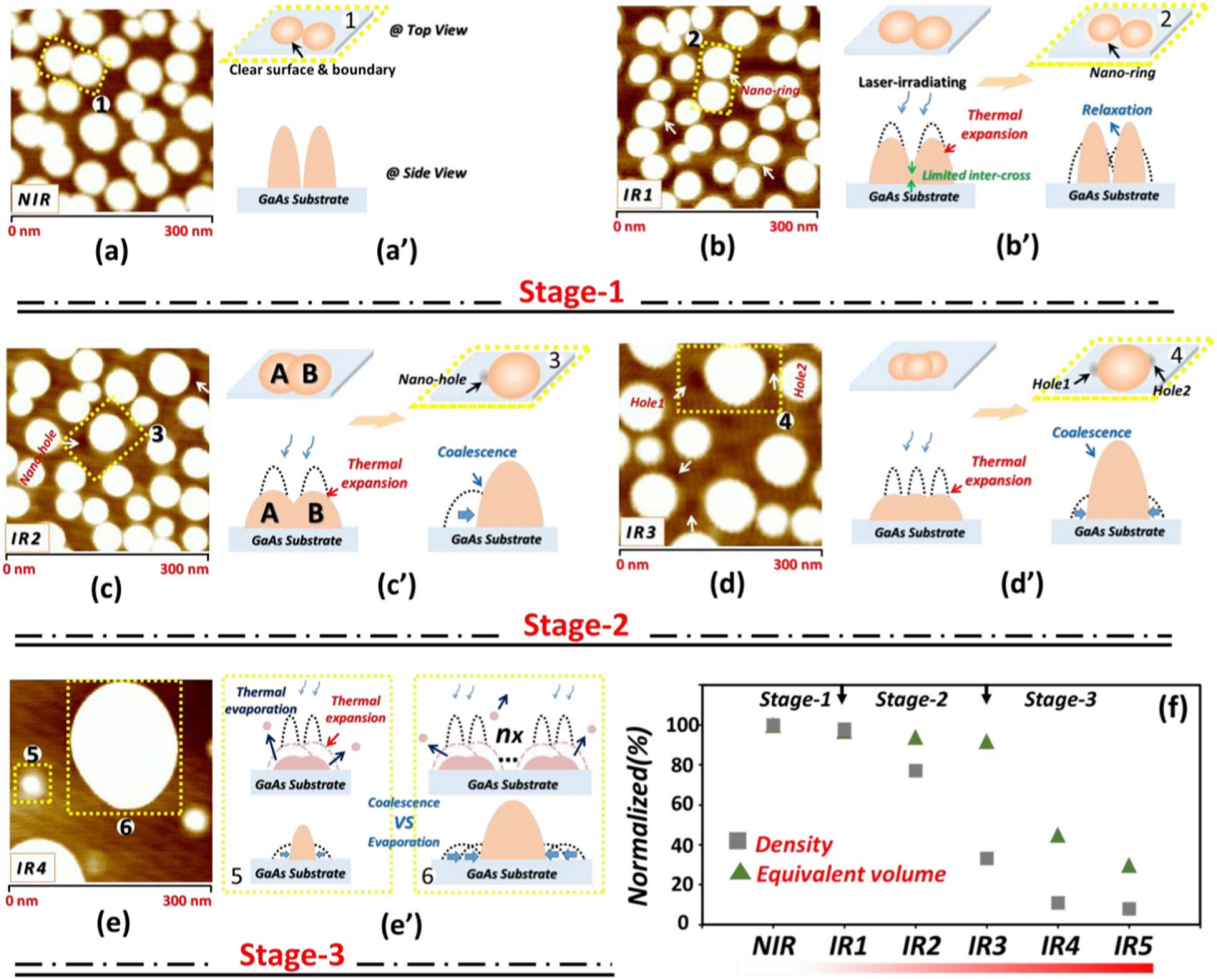
Magnifications of AFM morphology result respectively regarding **a** *NIR* and **b**–**e** *IR1-IR4* and the corresponding drawings of morphology dynamics for (**a**′) *NIR* and (**b**′–**e**′) *IR1-IR4*, for a convenient discuss, droplets with the typical morphology property of each magnification are carefully selected and marked by yellow dotted rectangles; **f** Normalized density and equivalent volume results of the droplets in Fig. 1a/*NIR*, Fig. 1b/*IR1*, Fig. 1c/*IR2*, Fig. 1d/*IR3*, Fig. 1e/*IR4* and Fig. 1f/*IR5*

In order to interpret the above experimental data, firstly, five partial areas selected from *NIR* and *IR1-IR4* are enlarged and illustrated in Fig. 2a–e, respectively. Secondly, we additionally calculated the equivalent volume (*EV*) of the Ga-droplets in *NIR* and *IR1-IR5*. In the calculation, the section profile of Ga droplet is approximately assumed as the mode of spherical cap [26], then the volume of each droplet can be given by

1 $${\text{V}} =\uppi {\text{r}}^{3} \left( {2 - 3\cos\uptheta + \cos^{3}\uptheta } \right)/3\sin^{3}\uptheta$$

where *r* is the droplet radius and θ is the contact angle, respectively, at last the *EVs* for *NIR* and *IR1-IR5* were counted by summing up the volume of all the droplets in Fig. 1a–f correspondingly. Figure 2f shows the normalized *EV* results (triangles) and the normalized density data (squares) is included as well. Then the whole evolution of laser resizing could be divided into three stages: at the first stage (*NIR-IR1*): The original droplets in *NIR* (Fig. 2a) inter-stand very close and the surrounding of each droplet is clear and flat (see the drawing) which is sketched in Fig. 2a′. For *IR1* (wherein is irradiated by a quite low intensity), comparing with the *NIR*, the size distribution, density and *EV* are almost unchanged, but an emerging structure of nano-ring is observed to surround the droplets which is marked by the white arrows in Fig. 2b. We attribute it to the expansion of droplets induced by laser-heating. As is shown in Fig. 2b′, after the irradiation, the laser will heat the droplets to expand (the well-known thermal expansion). Whereas the expansion is not strong enough to make coalescence of the droplets due to the limited intercross. As the heat dissipated, the droplets will relax back to the original equilibrium state but leaving the trails of expansion which is shaped as a ring encircling the relaxed droplet (see the black arrow). Therefore in this stage, the *IRIT* is too weak to resizing the droplets; at the second stage (*IR2-IR3*): In Fig. 2c for *IR2*, the experimental evidence of the droplet coalescence is observed and pointed out with a yellow dotted rectangle. The marked droplet is neighbored by a nano-hole (white arrow) and it is much larger than any of *NIR* with the size of 70 nm wide and 12 nm high. This can be explained by the coalescence of two droplets as is shown in Fig. 2c′: for A_droplet_ and B_droplet_, with the *IRIT* increasing, the expansion is enhanced which results in more cross-over between them and then the more inter-cross will probably push A_droplet_ merging into B_droplet_ randomly thus leaves a nano-hole pre-drilled by A_droplet_ at the same time. While compared with *IR2*, in Fig. 2d for *IR3*, coalescence of three (see yellow dotted rectangle/Fig. 2d′) or even more droplets are discovered which reflects a more strong effect of laser resizing. Hence, for *IR2* and *IR3*, the statistical data of size distribution and density could be explained as a result of the coalescence. Furthermore, as is seen in Fig. 2f, both of *IR2* and *IR3* still keep the same *EV* level with *NIR* in contrast to the sharp reduction of density. That means at this stage, the laser-shooting only resizes the droplets through thermal expansion without loss of Ga atoms. However, at the third stage of *IR4-IR5*: the *EV* of droplets begins to decrease sharply. It indicates that the *LIR* will not only expand the droplets but also accompanying with thermal evaporation of Ga atoms. Once the *IRIT* exceeds to a certain value, the pulsed laser may instantaneously heat the droplet to over the evaporation threshold of Ga. So the resizing of droplets in this stage is co-governed by coalescence and evaporation. Figure 2e′ illustrates the interplay: if the coalescence not compensate the loss of Ga by thermal evaporation, the droplet size will shrink (see the mini droplet marked in Fig. 2e) and otherwise, it will increase. Especially, some huge droplets may be produced (see the huge droplet marked in Fig. 2e) by coalescence of multi-droplets under certain probability. Then this kind of competition can well explain why the size-shift of *IR4* and *IR5* specially broadens to both sides. So far, resizing of the droplets by in-situ pulsed *LIR* has been well investigated in the perspectives of both performance and principle. To make the work more well-organized, in the following we have carried out another two designed experiments.

**Fig. 3:**
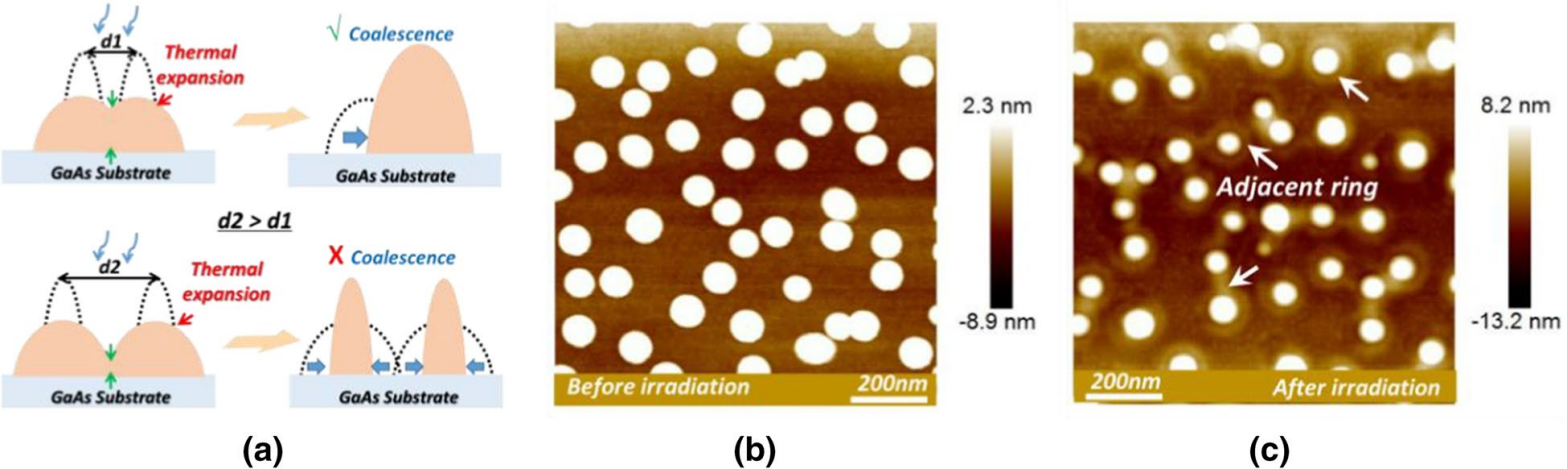
**a** Graphic illustration of the effect of inter-distance on the coalescence between two droplets; AFM morphology results of the droplets grown at 280 °C **b** before and **c** after irradiation

On the one hand, according to the principle of our explanation of thermal-expansion induced coalescence, besides the *IRIT*, the inter-droplet distance, i.e., the density of droplet, is the other key parameter. As is shown in Fig. 3a, if we separate two droplets in a larger gap (from d1 to d2), the coalescence should be relatively inhibited resulting from the reduced inter-cross during the same thermal expansion. Hence, we prepared a new droplet sample at the temperature of 280 °C. As the temperature increased, the droplet density of *NIR* (Fig. 3b) is rapidly decrease to 5 × 10^9^/cm^2^, nearly 1/8 of the sample at 180 °C and the interspace among droplets has been amplified effectively. After irradiation, as is seen in Fig. 3c, the droplets still have the equal density to the *NIR* but are encircled by very remarkable adjacent rings (see white arrows). It reflects that the coalescence is indeed prevented even with strong thermal expansion and thus further solidifies our explanation powerfully.

**Fig. 4:**
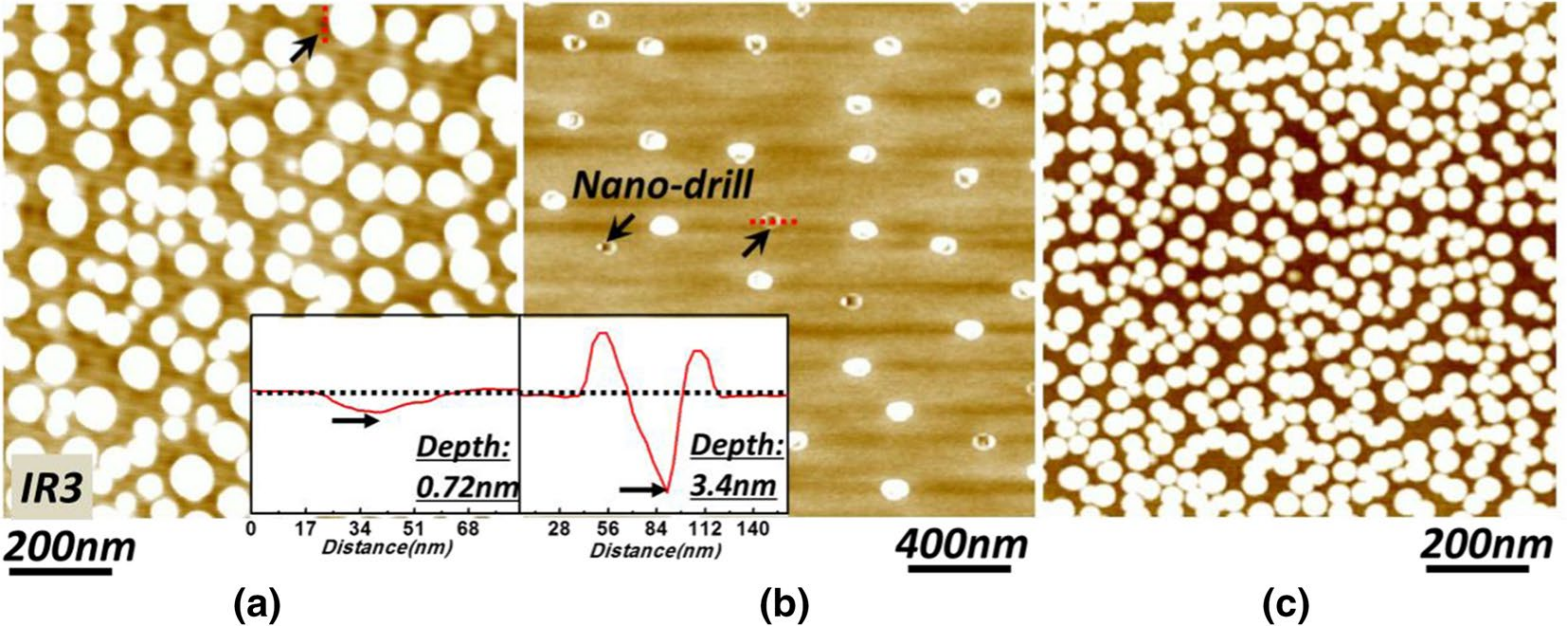
AFM morphology results of the droplets in **a** *IR3*, **b** the droplets grown at 350 °C followed by an anneal of 2 min at the same temperature and **c** the droplets grown at 180 °C followed by an anneal of 15 min at the same temperature

On the other hand, it is worth pointing out that the nano-holes (Fig. 4a) observed in our work are very shallow with depths of sub-nanometer (less than three atomic layers)(see the inset). So impressively, the drill effect of droplets is strictly suppressed and could be almost neglected which benefits from the low Sub temperature. To present the potential risk of nano-drill as the temperature increasing for the droplets, we fabricated another sample at a high temperature of 350 °C. After the growth finished, the Sub temperature didn’t decrease immediately but with a short interrupt of only 2 min before rapid cooling down. Figure 4b shows the morphology result, we could see a serious drill effect has happened and it has destroyed the droplets badly. And some droplets (see arrows) are even completely eroded up and replaced by nano-holes with etching depth of several nanometers (see the inset). On the contrary, as is suggested in Fig. 4c, the droplets prepared at 180 °C can still keep stable after an interrupt as long as 15 min.

## Conclusions

In conclusion, we have conducted a research on MBE in situ shooting on the Ga-droplets at 180 °C by pulsed laser and demonstrated that the laser-shooting can facilely and high-efficiently adjust the size distribution of droplets. The morphology evolution of the droplet as a function of *IRIT* is carefully studied and the involved mechanism is also systematically clarified: for low irradiation level, the droplet size distribution is solely shifted to the large side which can be explained by the only effect of droplet-coalescence induced by laser-thermal expansion of the droplets; While for high irradiation level, the size-shift will specially extend to both sides and this is resulting from a kind of competition between coalescence and thermal-evaporation. So herein, we have reported a technology by using pulsed laser-irradiation to in situ resize the droplets at such a low temperature that can nearly prevent the droplets etching into the Sub. Apparently, our technology is compatible with the common droplet epitaxy solution beautifully with free of pollution, oxidation and damage. And what is worth mentioning is, by upgrading the mono-beam irradiation into muti-beam interference irradiation, that we can easily realize patterned-modification of the droplet size for a more controlled droplet epitaxy in the future.

## Data Availability

Not applicable.
